# Glaucumolides A and B, Biscembranoids with New Structural Type from a Cultured Soft Coral *Sarcophyton glaucum*

**DOI:** 10.1038/srep15624

**Published:** 2015-11-04

**Authors:** Chiung-Yao Huang, Ping-Jyun Sung, Chokkalingam Uvarani, Jui-Hsin Su, Mei-Chin Lu, Tsong-Long Hwang, Chang-Feng Dai, Shwu-Li Wu, Jyh-Horng Sheu

**Affiliations:** 1 Department of Marine Biotechnology and Resources, National Sun Yat-sen University, Kaohsiung 804, Taiwan; 2 National Museum of Marine Biology & Aquarium, Pingtung 944, Taiwan; 3 Institute of Marine Biotechnology, National Dong Hwa University, Pingtung 944, Taiwan; 4 Graduate Institute of Natural Products, School of Traditional Chinese Medicine, College of Medicine, Chang Gung University, Taoyuan 333, Taiwan; 5 Institute of Oceanography, National Taiwan University, Taipei 112, Taiwan; 6 General Study Center, National Kaohsiung Marine University, Kaohsiung 805, Taiwan; 7 Department of Medical Research, China Medical University Hospital, China Medical University, Taichung 404, Taiwan; 8 Graduate Institute of Natural Products, Kaohsiung Medical University, Kaohsiung 807, Taiwan

## Abstract

Glaucumolides A (**1**) and B (**2**), novel biscembranes composed of an unprecedented α,β-unsaturated ε-lactone, along with the known metabolites ximaolide A (**3**) and isosarcophytonolide D (**4**), were isolated from the cultured soft coral *Sarcophyton glaucum*. The structures of the new metabolites were determined by extensive spectroscopic analyses. Compounds **1** and **2** were shown to exhibit cytotoxicity against a limited panel of cancer cell lines. In anti-inflammation assay, compounds **1** and **2** displayed strong inhibition of superoxide anion generation and elastase release in human neutrophils stimulated by fMLP/CB. Furthermore, both **1** and **2** were shown to significantly inhibit the accumulation of the pro-inflammatory inducible nitric oxide synthase protein, and compounds **1**−**3** were found to effectively reduce the expression of cyclooxygenase-2 protein, in lipopolysaccharide-stimulated RAW264.7 macrophage cells.

Many cembrane-type diterpenes have been proven to exhibit cytotoxicity and anti-inflammatory activity^1^. More than 60 biscembranoids have been isolated from the soft corals of the genera *Sarcophyton*^2^, *Lobophytum*^12^, *Sinularia*^13^. In particular, those belonging to the genus *Sarcophyton* are the most prolific source of biscembranoids. A common structural feature among these biscembranes is biogenetically derived from Diels-Alder reaction of two different cembranoid units. In our continuing search for structurally unique bioactive metabolites from the genera *Sarcophyton*^14^, we carried out the chemical investigation of the cultured soft coral *Sarcophyton glaucum* (Fig. 1). This study has led to the discovery of two new Diels-Alder cyclized biscembranoids glaucumolides A and B (**1** and **2**), a known biscembranolide ximaolide A (**3**)^8^,^11^, along with isosarcophytonolide D (**4**)^9^, an expected biogenic dienophile precursor of **1** (Fig. 2). The structures of **1** and **2** were deduced by extensive spectroscopic analysis. Based on structural consideration, both **1** and **2** were classified as a new type of biscembranoids as they are biosynthesized using ε-lactonecembrane **6** as a new diene monomer which has not been discovered before the biosynthesis of biscembranoids formed by Diels-Alder reaction. The attracting biological activities of several known biscembranoids^13^,^15^ further prompted us to evaluate the cytotoxic and anti-inflammatory activities of isolated metabolites **1**–**4**. The results demonstrated that compounds **1** and **2**, possessing a γ- and an ε-lactone rings, exhibited inhibition against the growth of human cancer cell lines, promyelocytic leukemia (HL-60), leukemic lymphoblasts (CCRF-CEM), acute T lymphoblastic leukaemia (MOLT-4), and erythroleukemia (K-562), as well as anti-inflammatory activities by significantly reducing the superoxide anion generation and elastase release in human neutrophils stimulated by *N*-formyl-methionyl-leucyl-phenylalanine/cytochalasin B (fMLP/CB), and the expression of iNOS and COX-2 proteins in LPS-challenged RAW264.7 macrophage cells. In contrast, without the presence of the mentioned lactone rings, **3** only displayed weaker inhibition on COX-2 accumulation in the same macrophage cells.

## Results

**Figure 3:**
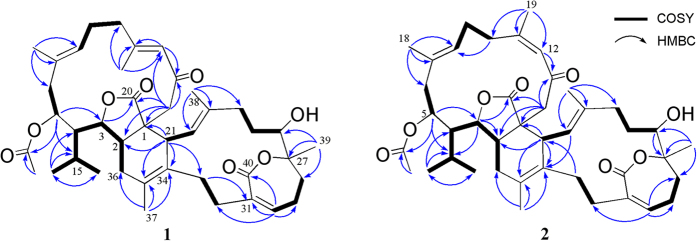
Selected COSY (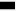) and HMBC (→) correlations of 1 and 2.

Glaucumolide A (**1**), [α]^25^_D_ −207 (*c* 0.007, CHCl_3_), was isolated as an amorphous solid. Its molecular formula, C_42_H_58_O_8_, was established by HRESIMS (713.4022 *m/z*, [M + Na]^+^), implying 14 degrees of unsaturation. The presence of the hydroxyl group was suggested by an absorption band at 3499 cm^–1^ in the IR spectrum. The ^13^C NMR spectroscopic data of **1** (Table 1) showed the presence of 42 carbon atoms, including eight methyls, 11 methylenes, 11 methines, and 12 quaternary carbons. Its NMR spectrum showed the signals of four vinyl methyls (*δ*_H_ 2.12, s; 1.81, s; 1.65, s; 1.62, s; *δ*c 20.2, 19.6, 17.9, 16.0), one methyl attached to oxygen-bearing quaternary carbon (*δ*_H_ 1.32, s; *δ*c 22.0), one acetoxy group (*δ*_H_ 2.13, s; *δ*c 170.5, C; 21.5, CH_3_), two methyls of an isopropyl group (*δ*_H_ 2.20, m; 1.10, d, *J* = 6.8 Hz; 1.08, d, *J* = 6.8 Hz; *δ*c 25.7, CH; 25.1, CH_3_; 18.2, CH_3_); four trisubstituted double bonds (*δ*_H_ 5.93, br s; 5.78, s; 5.14, dd, *J* = 6.4, 6.4 Hz; 5.11, d, *J* = 10.0 Hz; *δ*c 161.1, C; 138.7, C; 135.1, CH; 132.8, C; 132.5, C; 127.2, CH; 125.0, CH; 123.0, CH); one tetrasubstituted double bond (*δ*c 132.1, C; 127.4, C); three oxygen-bearing methines (*δ*_H_ 4.82, dd, *J* = 11.2, 3.2 Hz; 4.16, dd, *J* = 7.6, 7.6 Hz; 3.95, dd, *J* = 11.2, 6.0 Hz; *δ*c 84.4, CH; 73.1, CH; 68.3, CH); one oxygenated quaternary carbon (*δ*c 82.9), and four carbonyl carbons (*δ*c 197.4, 177.9, 170.5, 168.9). These evidences indicated the possible presence of a biscembranoid skeleton in compound **1**. The COSY spectrum of **1** was used to identify seven different structural units from H_2_-36 to isopropyl protons H_3_-16 and H_3_-17 via H-2, H-3 and H-4; H-5 to H_2_-6; H-8 to H_2_-10; H-21 to H-22; H_2_-24 to H-26; H_2_-28 to H-30; and H_2_-32 to H_2_-33, which were further assembled by HMBC correlations H-5 to C-3 and C-15; H_2_-14 to C-1, C-2, C-12 and C-13; H_3_-16 and H_3_-17 to C-4 and C-15; H_3_-18 to C-6, C-7 and C-8; H_3_-19 to C-10, C-11 and C-12; H-21 to C-1, C-2, C-33, C-34 and C-35; H-30 to C-31, C-32 and C-40; H_3_-37 to C-34, C-35 and C-36; H_3_-38 to C-22, C-23 and C-24; and H_3_-39 to C-26, C-27 and C-28 (Fig. 3). Moreover, the HMBC correlations of H-3 and H_2_-14 to C-20 clearly suggested the presence of an α,β-unsaturated γ-lactone moiety at C-1–C-3 and C-20. Furthermore, the acetoxy group positioned at C-5 was confirmed from the HMBC correlations of H-5 (*δ* 4.82, dd, *J* = 11.2, 3.2 Hz) and protons of an acetate methyl (*δ* 2.13, s) to the ester carbonyl carbon at *δ* 170.5. In addition, in the ^13^C NMR spectrum of **1** the signal at *δ*c 68.3, and in the ^1^H NMR spectrum the signal at *δ*_H_ 4.16 (dd, *J* = 7.6, 7.6 Hz) could be attributed to a hydroxyl-bearing methine at C-26. On the base of above results and by considering the degrees of unsaturation and molecular formula, C-27 and C-40 should be linked by an oxygen atom to form an ε-lactone ring. The gross structure of **1** was thus established. Metabolite **1** is the first Diels-Alder cyclized biscembranoid possessing not only a saturated γ-lactone but also a seven-membered α,β-unsaturated ε-lactone in the molecule.

**Figure 4:**
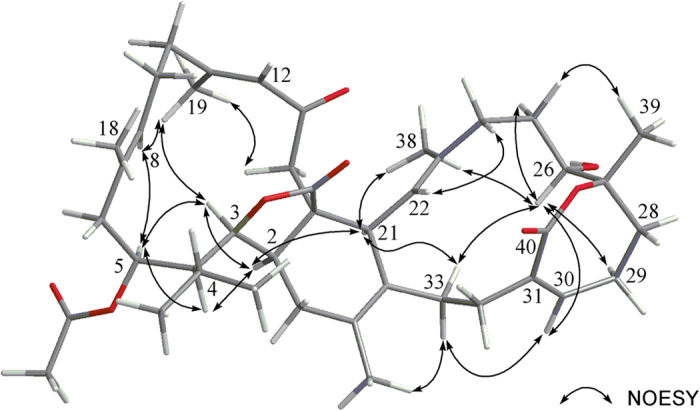
Selected NOE correlations for 1.

The relative configurations of the stereogenic centers in **1** were determined on the basis of NOE relationships and NMR spectroscopic data. It was found that NOESY correlations of H-3 (*δ* 3.95, dd, *J* = 11.2, 6.0 Hz) with H-2 (*δ* 2.33, m) and H-5 (*δ* 4.82, dd, *J* = 11.2, 3.2 Hz), H-4 (*δ* 1.50, d, *J* = 10.8 Hz) with H-2 and H-5, and H-2 with H-21 (*δ* 3.05, d, *J* = 10.0 Hz), and the upfield-shifted *δ*_H_ value of H-21α relative to the H-21β of related biscembranoids in ^1^H NMR^10^, indicated the α-orientation for H-2, H-3, H-4, H-5, and H-21. This observation was also strongly supported by the similar ^1^H NMR spectroscopic data of the above protons including chemical shifts and coupling constants with those of bislatumlide C^10^. Furthermore, the NOE correlations of H-21 with H-33α (*δ* 2.16, m) and H_3_-38, H_3_-38 with H-26 (*δ* 4.16, dd, *J* = 7.6, 7.6 Hz), and H-26 with H-25α (*δ* 1.82, m), H-29α (*δ* 2.46, m) and H-30 (*δ* 5.93, br s), reflected the α-orientations of H-26 and the R*-configuration at C-26. Above results and the NOE correlation between H_3_-39 (*δ* 1.32, s) and H-25β (*δ* 1.36, m) suggested the β-orientation of H_3_-39 and thus 27S* configuration of **1** as shown in Fig. 4. Further, the 26R* and 27S* configuration, not 26R* and 27R*, could be confirmed by comparing the *δ* values of C-7 (69.2) and C-8 (82.9) of known compound sartrolide C^16^ to the corresponding C-26 (68.3) and C-27 (82.9) of **1**, while sartrolide A^16^ with 26R* and 27R* configuration showed C-26 signal at *δ* 72.7 ppm. The chemical shift values of C-18 (17.9), C-19 (19.6) and C-38 (16.0) reflected the all *trans* geometry of the trisubstituted double bonds at C-7/C-8, C-11/C-12 and C-22/C-23 in the molecule of **1**. From the above observations, NOE correlation between H-30 and one of H_2_-33 (*δ* 2.66, m), and further analysis of other NOE interactions (Fig. 4), the relative configuration of **1** with rings A−E could be established.

Glaucumolide B (**2**), [α]^25^_D_ −221 (*c* 0.008, CHCl_3_), was showed the pseudomolecular ion peak [M + Na]^+^ at *m/z* 713.4023 in the HRESIMS, suggesting the molecular formula C_42_H_58_O_8_ and 14 degrees of unsaturation. The IR spectrum also suggested the presence of hydroxyl group in **2** (*ν*_max_ 3434 cm^–1^). The ^13^C NMR spectroscopic data (Table 1) of **2** were found to be resembled to those of **1**. Detailed analysis of 1D and 2D NMR spectra of **2** revealed the similar gross structure as that of **1**. However, it was found that H-12 (*δ* 5.98, s) showed significant NOE interaction with H_3_-19 (*δ* 1.89, s), and the signal of the C-19 in **2** was remarkably downfield-shifted (*δ* 19.6 in **1**, 24.8 in **2**), indicating a *Z* geometry of Δ^11(12)^ in **2**, in contrast to the 11*E* double bond in **1**. These results and other NMR data including NOE correlations, established the structure of compound **2** to be the 11*Z* isomer of **1**.

**Figure 5:**
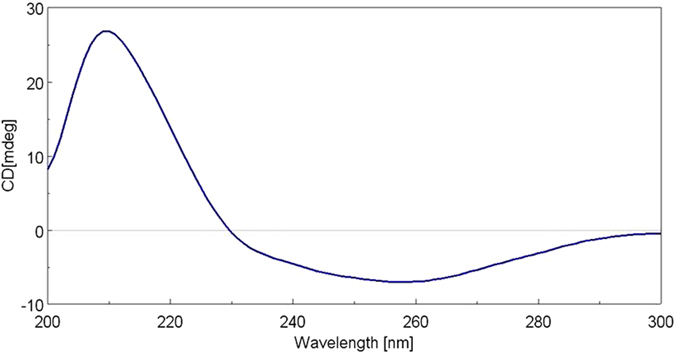
CD spectrum (1.2 × 10^−4^ M, MeOH) of 2.

The absolute configurations of **1** and **2** were further confirmed by comparison of the CD (circular dichroism) spectroscopic data with structurally related compound. As shown in Fig. 5, the CD spectrum of **2** showed a broad negative Cotton effect at 258 nm (*Δ*ε = −7.1) due to enone n–π* transition absorption while the intense positive cotton effect at 209 nm (*Δ*ε = +27.4) resulted from the π–π* transition of the two isolated Δ^22(23)^ and Δ^34(35)^ double bonds, very similar to that of bislatumlide C^10^. Thus, the absolute configurations for rings B and C should be the same for both bislatumlide C and **2**. One the basis of above results and the fact that both **1** and **2** were isolated from the same organism, the structures and absolute configurations of both **1** and **2** were found to possess the 1*S*,2*R*,3*S*,4*S*,5*S*,21*S*,26*R*,27*S*.

**Figure 6:**
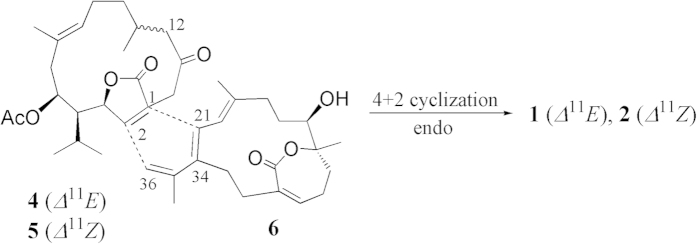
Plausible Diels-Alder reaction to derive compounds 1 and 2.

A plausible biosynthetic pathway, involving a key Diels–Alder reaction, was postulated for the biosynthesis of compounds **1** and **2** in Figure 6. It is obvious that **4** is one of the two precursors of **1**. Another proposed precursor should be the yet to be discovered compound **6**. [4+2] Endo cycloaddition of the dienophile **4** and diene **6** occurs between 1,2-*Z* double bond of **4** and ∆^21(34)^ and ∆^35(36)^ conjugated double bonds diene of **6**. Although the possible involvement of a biosynthetic Diels-Alder reaction to afford biscembranoid has been mentioned frequently, no any other bicembranoid was found to be formed by using ε-lactone cembrane **6** or related cembranoidal ε-lactone as the diene precursor. In addition, almost all the previous cembrane dimers exhibited the reactive dienophile double bond at positions C-1 and C-14 (according to the numbering assigned to compound **4**), whereas the dienophile double bond in **4** was located at C-1 and C-2. The Diels–Alder addition which arises from supra–supra transition state explains the *trans* stereochemistry of H-2 and lactone as well as the *cis* geometry of lactone and H-21. Analogously, the formation of biscembrane **2** by Diels–Alder cyclization of the 11*Z* isomer of **4**, sarcophytonolide A (**5**)^6^,^9^, with compound **6** could be hypothesized.

The cytotoxicity of compounds **1**–**4** against four human cancer cell lines, HL-60, CCRF-CEM, MOLT-4, and K-562 was investigated. The results (Table 2) demonstrated that compound **2** exhibited significant cytotoxicity against HL-60 and CCRF-CEM cancer cell lines with ED_50_ values of 3.8 ± 0.9 and 5.3 ± 1.4 *μ*g/mL, respectively. Also, compound **1** exhibited cytotoxicity against the above two cell lines with ED_50_ values of 6.6 ± 1.2 and 7.4 ± 1.5 *μ*g/mL, respectively. Further, compounds **1** and **2** displayed weaker activity against MOLT-4 and K-562 cell lines (ED_50_ 11.0–19.2 *μ*g/mL). In contrast, compound **3** was inactive toward all the tested cell lines. Perhaps the enhanced cytotoxicity of compounds **1** and **2** relative to **3** is owing to the presence of a α,β-unsaturated ε-lactone ring.

The anti-inflammatory activities of compounds **1**–**4** on neutrophil pro-inflammatory responses were evaluated by measuring their ability in suppressing fMLP/CB-induced superoxide anion (O_2_^− •^) generation and elastase release in human neutrophils, and the results were shown in Table 3. From the results, **1** and **2** showed strong inhibitions (88.42 ± 3.97 and 91.75 ± 3.08%, respectively.) toward superoxide anion generation at 10 μM. Both of them also exhibited potent inhibitory activity against elastase release, with 88.94 ± 6.96 and 103.25 ± 1.89% inhibitions in the same fMLP/CB-stimulated cells at the same concentration. The IC_50_ values of **1** and **2** in inhibiting the superoxide generation and elastase release were also measured. Although compound **4** did not exhibit strong activity in inhibiting superoxide anion generation, it was shown to display significant inhibitory activity in elastase release.

**Figure 7:**
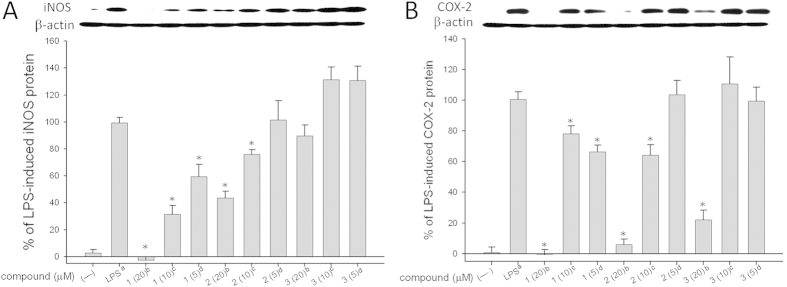
Effect of compounds 1–3 on iNOS and COX-2 protein expression of RAW264.7 macrophage cells by immunoblot analysis. (**A**) Immunoblots of iNOS and β-actin; (**B**) Immunoblots of COX-2 and β-actin. The values are mean ± SEM (*n* = 3). Relative intensity of the LPS alone stimulated group was taken as 100%. Under the same experimental condition CAPE (caffeic acid phenylethyl ester, 10 *μ*M) reduced the levels of the iNOS and COX-2 to 2.5 ± 3.7% and 50.3 ± 8.7%, respectively. *Significantly different from LPS alone stimulated group (*p < 0.05). ^*a*^Stimulated with LPS. ^*b*^Stimulated with LPS in the presence of **1**–**3** (20 *μ*M). ^*c*^Stimulated with LPS in the presence of **1**–**3** (10 *μ*M). ^*d*^Stimulate with LPS in the presence of **1**–**3** (5 *μ*M).

The *in vitro* anti-inflammatory activity of compounds **1**–**3** was also studied. In this assay, the up-regulation of the proinflammatory iNOS and COX-2 proteins of LPS-stimulated RAW264.7 macrophage cells was evaluated using immunoblot analysis. The results (Fig. 7) showed that at concentrations of 5, 10, and 20 *μ*M, compound **1** was found to significantly reduce the levels of iNOS and COX-2 to 59.4 ± 9.0 and 66.5 ± 4.4%; 31.3 ± 6.5 and 78.3 ± 5.0%; and −2.6 ± 2.7 and −0.5 ± 3.2%, respectively. At concentrations of 10 and 20 *μ*M, compound **2** was found to significantly reduce the levels of iNOS and COX-2 to 75.9 ± 3.5 and 64.3 ± 6.9%; and 43.4 ± 5.0 and 6.0 ± 3.6%, respectively. Moreover, at 20 *μ*M, **3** also reduced the level of COX-2 expression to 22.0 ± 6.5% in macrophage cells with LPS treatment. As they did not exhibit cytotoxicity to RAW264.7 cells, they might be promising anti-inflammatory agents. Also, **2** possessing promising cytotoxicity, could become a candidate for future anticancer drug development.

## Discussion

Compounds **1** and **2** are structurally novel as they belong to a new type of biscembranoids using the not yet isolated ε-lactonecembrane **6** as the first time discovered diene precursor for the biosynthesis of biscembranoids by Diels-Alder reaction. Metabolites **1** and **2**, with the presence of a α,β-unsaturated ε-lactone ring, were shown to exhibit cytotoxicity against a limited panel of HL-60, CCRF-CEM, MOLT-4 and K-562 cancer cell lines. Compounds **1** and **2** also exhibited potent anti-inflammatory activity in inhibiting the superoxide generation and elastase release in fMLP/CB-induced human neutrophils. Furthermore, both **1** and **2** were shown to significantly inhibit the accumulation of the pro-inflammatory inducible nitric oxide synthase protein, and compounds **1**–**3** were found to effectively reduce the expression of cyclooxygenase-2 protein, in lipopolysaccharide-stimulated RAW264.7 macrophage cells.

## Conclusion

The unusual structural framework with α,β-unsaturated ε-lactone system were reported here for glaucumolides A and B (**1** and **2**), along with a known biscembranolide ximaolide A (**3**), and isosarcophytonolide D (**4**) from the cultured soft coral *S. glaucum*. From the results of biological activities, it appears that compounds **1** and **2** might be useful for future biomedical applications. The discovery of glaucumolides with a novel carbon scaffold provides additional evidence that cultured soft corals might be a promising source of structurally novel bioactive natural products which could be used for further pharmacological investigation.

## Methods

### General Experimental Procedures

Optical rotations were measured on a Horiba High Sensitivity Polarimeter SEPA-300. Ultraviolet spectra were recorded on a JASCO V-650 spectrophotometer. IR spectra were recorded on a JASCO FT/IR-4100 infrared spectrophotometer. CD spectra were recorded on a JASCO J-815 CD spectrophotometer. NMR spectra were recorded on a Varian 400MR FT-NMR instrument at 400 MHz for ^1^H and 100 MHz for ^13^C in CDCl_3_. LRMS and HRMS were obtained by ESI on a Bruker APEX II mass spectrometer. Silica gel (Merck, 230–400 mesh) was used for column chromatography. Precoated silica gel plates (Merck, Kieselgel 60 F-254, 0.2 mm) were used for analytical TLC. High-performance liquid chromatography was performed on a Hitachi L-2455 HPLC apparatus with a Supelco C18 column (250 × 21.2 mm, 5 μm).

### Animal Material

The cultured soft coral *Sarcophyton glaucum* used in this study was originally collected from the wild and cultured for five years in an 80-ton cultivation tank (height 1.6 m) located in the National Museum of Marine Biology and Aquarium, Taiwan. The tank was a semiclosed recirculating aquaculture system and did not require deliberate feeding. To the best of our knowledge, this is the first farming system for *S. glaucum* in the world. The specimens were then collected by hand in January 2010 and were stored in a −20 °C freezer. The soft coral was identified by one of the authors (C.-F.D.). A voucher specimen (specimen no. 201001C3) was deposited in the Department of Marine Biotechnology and Resources, National Sun Yat-sen University.

### Extraction and Isolation

The frozen bodies of *S. glaucum* (0.6 kg, wet wt) were minced and extracted exhaustively with CH_2_Cl_2_ and MeOH (1:1, 0.5 L × 6). The CH_2_Cl_2_ and MeOH extract of the soft coral *S. glaucum* was partitioned between EtOAc and H_2_O to afford the EtOAc-soluble fraction. The EtOAc extract (4.5 g) was chromatographed over silica gel by column chromatography and eluted with EtOAc in *n*-hexane (0–100%, stepwise) and then with MeOH in EtOAc (5–50%, stepwise) to yield 24 fractions. Fraction 15 (23.3 mg), eluting with *n*-hexane–EtOAc (5:1), was further purified by reversed-phase HPLC using MeOH−H_2_O (3:1) to afford **4** (2.6 mg). Fraction 20 (69.7 mg), eluting with *n*-hexane–EtOAc (1:1), was further purified over silica gel using *n*-hexane–acetone (2:1) to afford six subfractions (A1–A6). Subfraction A3 (13.5 mg) was further purified by reversed-phase HPLC using MeOH−H_2_O (5:2) to afford **3** (5.2 mg). Subfraction A4 (20.0 mg) was further purified by reversed-phase HPLC using MeOH−H_2_O (2:1) to afford **1** (4.4 mg) and **2** (2.8 mg).

### Glaucumolide A (1)

white amorphous powder; [α]^25^_D_ −207 (*c* 0.007, CHCl_3_); IR (neat) ν_max_ 3499, 2939, 2876, 1734, 1716, 1699, 1375, 1239, 1024, and 755 cm^−1^; UV (MeOH) λ_max_ (log *ε*) 205 (3.4) and 236 (3.2) nm; CD (1.9 × 10^−4^ M, MeOH) λ_max_ (*Δ*ε) 244 (−23.7), and 215 (+36.0) nm; ^13^C and ^1^H NMR data, see Table 1; ESIMS *m/z* 713 [M + Na]^+^; HRESIMS *m/z* 713.4022 [M + Na]^+^ (calcd for C_42_H_58_O_8_Na, 713.4024).

### Glaucumolide B (2)

white amorphous powder; [α]^25^_D_ −221 (*c* 0.008, CHCl_3_); IR (neat) ν_max_ 3434, 2940, 2878, 1734, 1716, 1698, 1376, 1239, 1024, and 754 cm^−1^; UV (MeOH) λ_max_ (log *ε*) 205 (3.3) and 236 (3.2) nm; CD (1.2 × 10^−4^ M, MeOH) λ_max_ (*Δ*ε) 258 (−7.1), and 209 (+27.4) nm; ^13^C and ^1^H NMR data, see Table 1; ESIMS *m/z* 713 [M + Na]^+^; HRESIMS *m/z* 713.4022 [M + Na]^+^ (calcd for C_42_H_58_O_8_Na, 713.4023).

### Cytotoxicity Testing

Cell lines were purchased from the American Type Culture Collection (ATCC). Cytotoxicity assays of compounds **1**–**4** were performed using the Alamar Blue assay^18^,^19^. To measure the cytotoxicity activities of tested compounds, three concentrations in DMSO with three replications were performed on each cell line. 5-Fluorouracil and DMSO were used as positive and negative controls, respectively in this assay.

### Preparation of Human Neutrophils

Human neutrophils obtained from peripheral blood of healthy adult volunteers (20–30 years old) were enriched using a standard method of dextran sedimentation, Ficoll-Hypaque centrifugation, and hypotonic lysis^20^,^21^. Purified neutrophils were resuspended in a Ca^2+^ -free HBSS buffer (pH 7.4) at 4 °C prior to use.

### Measurement of O2^− •^ Generation

The O_2_^**− •**^ production was assayed based on the superoxide oxide dismutase inhibitable reduction of ferricytochrome *c*^22^. Briefly, neutrophils (6 × 10^5^ cells/mL) incubated with ferricytochrome *c* (0.5 mg/mL) and Ca^2+^ (1 mM) were equilibrated at 37 °C for 2 min and then treated with DMSO as control or different concentrations of compounds for 5 min. Neutrophils were activated by 100 nM fMLP for 10 min in the pretreatment of cytochalasin B (CB, 1 μg/mL) for 3 min (fMLP/CB).

### Measurement of Elastase Release

The elastase release was assayed using MeO-Suc-Ala-Ala-Pro-Val-*p*-nitroanilide as the enzyme substrate^23^,^24^. Briefly, neutrophils (6 × 10^5^ cells/mL) incubated with MeO-Suc-Ala-Ala-Pro-Val-*p*-nitroanilide (100 μM) were equilibrated at 37 °C for 2 min and treated with compounds for 5 min. Neutrophils were then activated with fMLP (100 nM)/CB (0.5 μg/mL) for 10 min.

### Statistical Analysis

Results are expressed as the mean ± SEM, and comparisons were made using Student’s t-test. A probability value of 0.05 or less was considered significant. The software SigmaPlot was used for the statistical analysis.

### *In Vitro* Anti-Inflammatory Assay

Macrophage (RAW264.7) cells were purchased from ATCC. *In vitro* anti-inflammatory activities of compounds **1**–**3** were measured by examining the inhibition of lipopolysaccharide (LPS) induced upregulation of iNOS (inducible nitric oxide synthetase) and COX-2 (cyclooxygenase-2) proteins in macrophages cells using Western blotting analysis^25^. For statistical analysis, all of the data were analyzed by a one-way analysis of variance (ANOVA), followed by the Student-Newman-Keuls *post hoc* test for multiple comparisons. A significant difference was defined as a *p* value of <0.05.

## Additional Information

**How to cite this article**: Huang, C.-Y. *et al.* Glaucumolides A and B, Biscembranoids with New Structural Type from a Cultured Soft Coral *Sarcophyton glaucum*. *Sci. Rep.* **5**, 15624; doi: 10.1038/srep15624 (2015).

## Supplementary Material

Supplementary Information

## Figures and Tables

**Figure 1:**
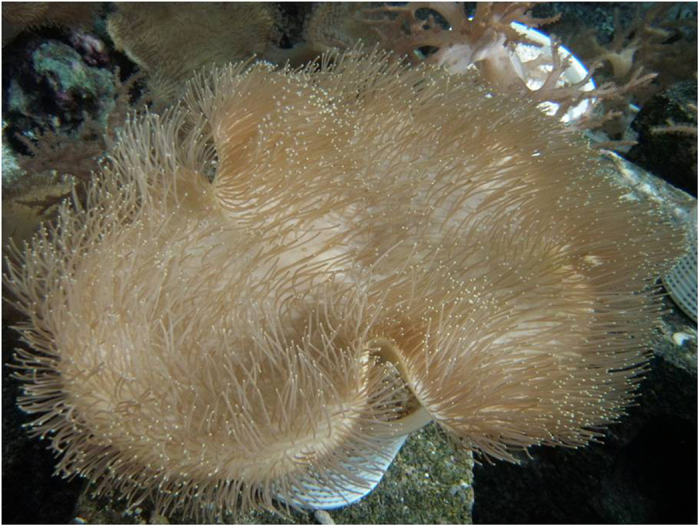
The cultured soft coral *Sarcophyton glaucum.*

**Figure 2:**
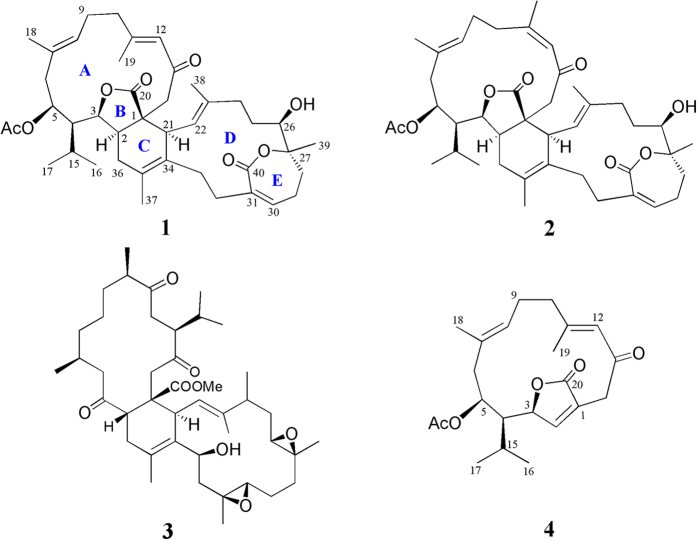
Chemical structures of metabolites 1–4.

**Table 1:** ^1^H and ^13^C NMR Data of 1 and 2.

Position	1		2	
	^1^H^a^	^13^C^b^	^1^H^a^	^13^C^b^
1		53.3 (C)		52.9 (C)
2	2.33 m	41.5 (CH)^d^	1.87 m	41.4 (CH)
3	3.95 dd (11.2, 6.0)^c^	84.4 (CH)	4.01 dd (11.2, 5.6)	85.5 (CH)
4	1.50 d (10.8)	48.7 (CH)	1.85 m	47.3 (CH)
5	4.82 dd (11.2, 3.2)	73.1 (CH)	4.82 dd (11.2, 3.2)	71.9 (CH)
6	2.28 m; 2.24 m	41.0 (CH_2_)	2.26 m; 2.18 m	41.7 (CH_2_)
7		132.5 (C)		131.7 (C)
8	5.14 dd (6.4, 6.4)	127.2 (CH)	5.17 dd (7.6, 7.6)	127.9 (CH)
9	2.32 m	24.7 (CH_2_)	2.29 m; 2.21 m	25.1 (CH_2_)
10	2.31 m; 2.25 m	40.6 (CH_2_)	3.00 m	31.2 (CH_2_)
11		161.1 (C)		162.7 (C)
12	5.78 s	125.0 (CH)	5.98 s	124.6 (CH)
13		197.4 (C)		198.1 (C)
14	2.79 d (13.2); 2.46 d (13.2)	50.7 (CH_2_)	2.99 m; 2.37 m	52.4 (CH_2_)
15	2.20 m	25.7 (CH)	2.17 m	25.6 (CH)
16	1.08 d (6.8)	18.2 (CH_3_)	1.08 d (6.8)	18.4 (CH_3_)
17	1.10 d (6.8)	25.1 (CH_3_)	1.17 d (6.8)	25.0 (CH_3_)
18	1.65 s	17.9 (CH_3_)	1.65 s	17.1 (CH_3_)
19	2.12 s	19.6 (CH_3_)	1.89 s	24.8 (CH_3_)
20		177.9 (C)		179.1 (C)
21	3.05 d (10.0)	45.5 (CH)	2.96 m	45.1 (CH)
22	5.11 d (10.0)	123.0 (CH)	4.94 d (10.8)	122.8 (CH)
23		138.7 (C)		138.3 (C)
24	2.12 m; 2.08 m	36.2 (CH_2_)	2.14 m; 2.02 m	36.4 (CH_2_)
25	1.82 m; 1.36 m	29.1 (CH_2_)	1.84 m; 1.31 m	29.2 (CH_2_)
26	4.16 dd (7.6, 7.6)	68.3 (CH)	4.16 dd (7.6, 7.6)	67.8 (CH)
27		82.9 (C)		82.6 (C)
28	2.18 m; 2.04 m	34.8 (CH_2_)	2.20 m; 2.04 m	34.6 (CH_2_)
29	2.46 m	26.2 (CH_2_)	2.47 m	26.6 (CH_2_)
30	5.93 br s	135.1 (CH)	5.89 br s	135.4 (CH)
31		132.8 (C)		ND^e^
32	2.58 m; 2.18 m	30.0 (CH_2_)	2.65 m; 2.35 m	30.1 (CH_2_)
33	2.66 m; 2.16 m	29.1 (CH_2_)	2.79 m; 2.07 m	29.3 (CH_2_)
34		132.1 (C)		131.3 (C)
35		127.4 (C)		127.4 (C)
36	2.31 m; 2.04 m	34.8 (CH_2_)	2.31 m; 2.10 m	35.3 (CH_2_)
37	1.81 s	20.2 (CH_3_)	1.81 s	20.3 (CH_3_)
38	1.62 s	16.0 (CH_3_)	1.60 s	15.9 (CH_3_)
39	1.32 s	22.0 (CH_3_)	1.31 s	21.8 (CH_3_)
40		168.9 (C)		168.5 (C)
41	2.13 s	21.5 (CH_3_)	2.11 s	21.5 (CH_3_)
42		170.5 (C)		170.6 (C)

^a^Recorded at 400 MHz in CDCl_3_ at 25 °C.

^b^Recorded at 100 MHz in CDCl_3_ at 25 °C.

^c^*J* values (Hz) in parentheses.

^d^Attached protons were deduced by DEPT experiment.

^e^Designate signal not detected^17^.

**Table 2:** Cytotoxicity of compounds 1–4.

cancer cell line	Compounds (ED_50_, *μ*g/mL)				
	1	2	3	4	5-Fluorouracil
HL-60	6.6 ± 1.2	3.8 ± 0.9	–a	13.0 ± 1.9	10.7 ± 0.5
CCRF-CEM	7.4 ± 1.5	5.3 ± 1.4	–	15.3 ± 2.5	2.3 ± 0.6
MOLT-4	11.0 ± 2.8	11.0 ± 2.2	–	17.2 ± 3.1	0.9 ± 0.2
K-562	19.2 ± 2.3	12.6 ± 0.7	–	–	4.3 ± 1.2

The values are mean ± SEM (*n* = 3).

^a^>20 *μ*g/mL

**Table 3:** Inhibitory effects of compounds 1–4 on superoxide anion generation and elastase release by human neutrophils.

Compound	Superoxide anion			Elastase release		
	IC^50^ (μM)^b^	Inh %^a^		IC^50^ (μM)	Inh %	
1	2.79 ± 0.66	88.42 ± 3.97	***	3.97 ± 0.10	88.94 ± 6.96	***
2	2.79 ± 0.32	91.75 ± 3.08	***	3.97 ± 0.10	103.25 ± 1.89	***
3	>10	15.33 ± 4.15	*	>10	15.13 ± 3.58	*
4	>10	12.40 ± 2.56	**	>10	27.12 ± 3.08	***
Idelalisib	0.07 ± 0.01	102.81 ± 2.21	***	0.28 ± 0.09	99.56 ± 4.19	***

^a^Percentage of inhibition (Inh %) at 10 μM concentration. Results are presented as mean ± S.E.M. (n = 3). *P < 0.05, **P < 0.01, ***P < 0.001 compared with the control value.

^b^Concentration necessary for 50% inhibition (IC^50^).
