# Supplementary material for: Glaucumolides A and B, Biscembranoids with New Structural Type from a Cultured Soft Coral Sarcophyton glaucum
Source: Sci Rep. 2015 Nov 4;5:15624. doi: 10.1038/srep15624 (PMC4632109; doi:10.1038/srep15624)
Supplement: Supplementary Information [file srep15624-s1.pdf]

Supplementary Information

Glaucumolides A and B, Biscembranoids with New Structural Type from a Cultured Soft Coral *Sarcophyton glaucum*

Chiung-Yao Huang, Ping-Jyun Sung, Chokkalingam Uvarani, Jui-Hsin Su, Mei-Chin Lu, Tsong-Long Hwang, Chang-Feng Dai, Shwu-Li Wu and Jyh-Horng Sheu*

Corresponding author: sheu@mail.nsysu.edu.tw

List of Contents

S1. HREIMS spectrum of **1** P2

S2. ^{1}H NMR spectrum of **1** in $CDCl_3$ P3

S3. ^{13}C NMR spectrum of **1** in $CDCl_3$ P4

S4. DEPT spectra of **1** in $CDCl_3$ P5

S5. HSQC spectrum of **1** in $CDCl_3$ P6

S6. COSY spectrum of **1** in $CDCl_3$ P7

S7. HMBC spectrum of **1** in $CDCl_3$ P8

S8. NOESY spectrum of **1** in $CDCl_3$ P9

S9. CD spectrum (1.9×10^{-4}, MeOH) of **1**. P10

S10. HREIMS spectrum of **2** P11

S11. ^{1}H NMR spectrum of **2** in $CDCl_3$ P12

S12. ^{13}C NMR spectrum of **2** in $CDCl_3$ P13

S13. DEPT spectra of **2** in $CDCl_3$ P14

S14. HSQC spectrum of **2** in $CDCl_3$ P15

S15. COSY spectrum of **2** in $CDCl_3$ P16

S16. HMBC spectrum of **2** in $CDCl_3$ P17

S17. NOESY spectrum of **2** in $CDCl_3$ P18

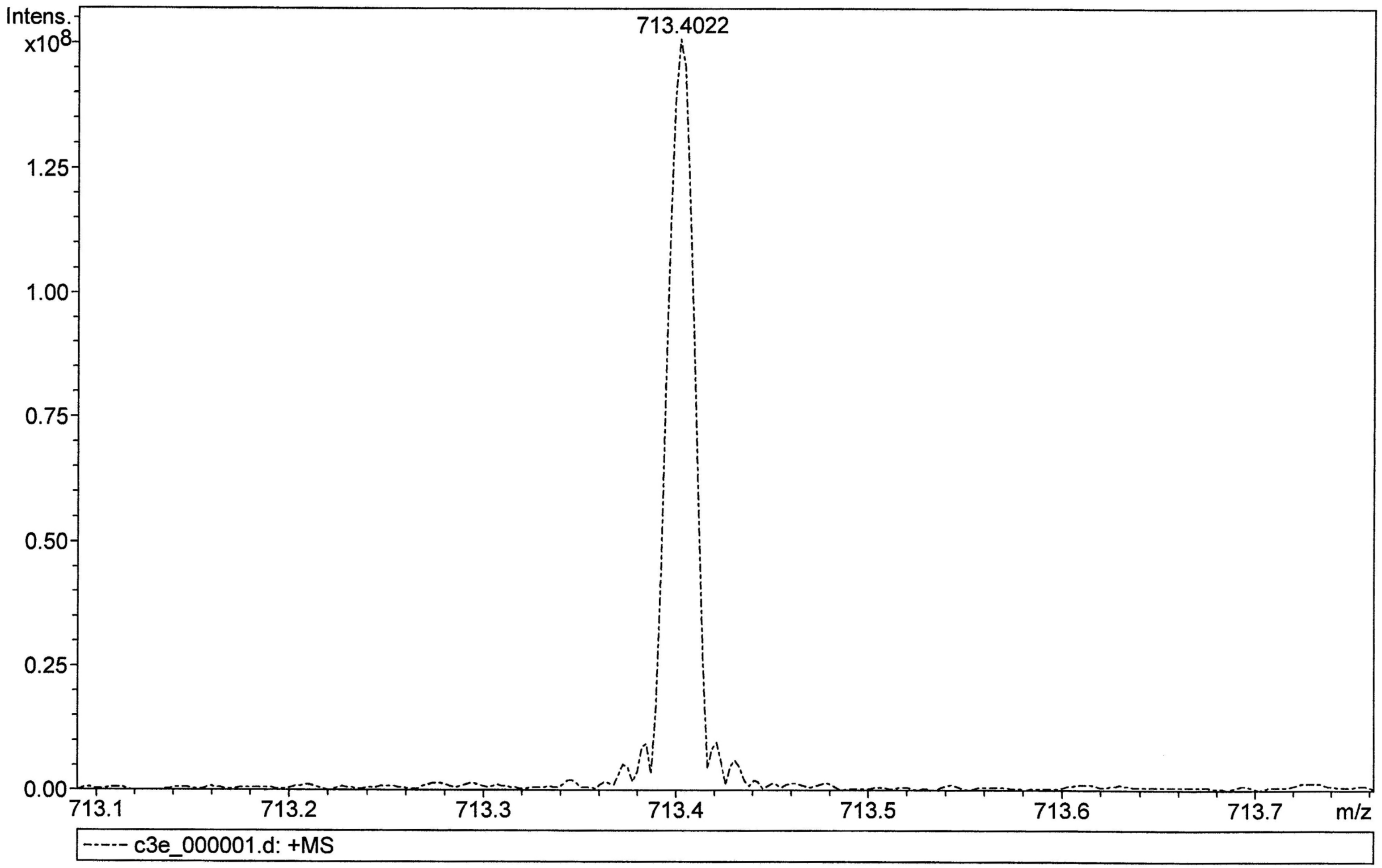

Meas. m/z	#	Formula	Score	m/z	err [mDa]	err [ppm]	mSigma	rdb	e¯ Conf	N-Rule
713.4022	1	C 42 H 58 Na O 8	95.14	713.4024	0.2	0.2	3.3	13.5	even	ok

S1. HREIMS spectrum of **1**

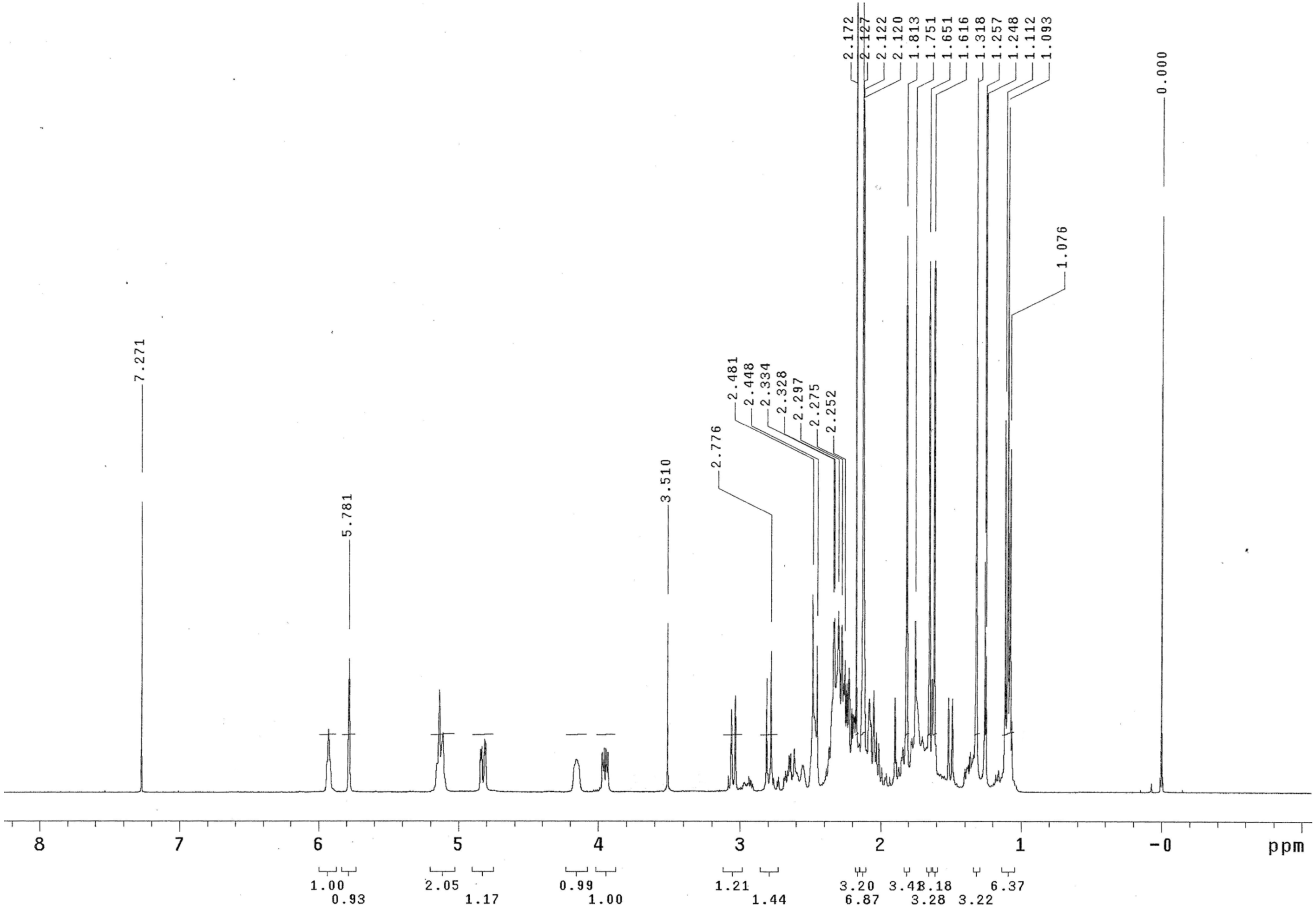

S2. ^{1}H NMR spectrum of **1** in $CDCl_3$

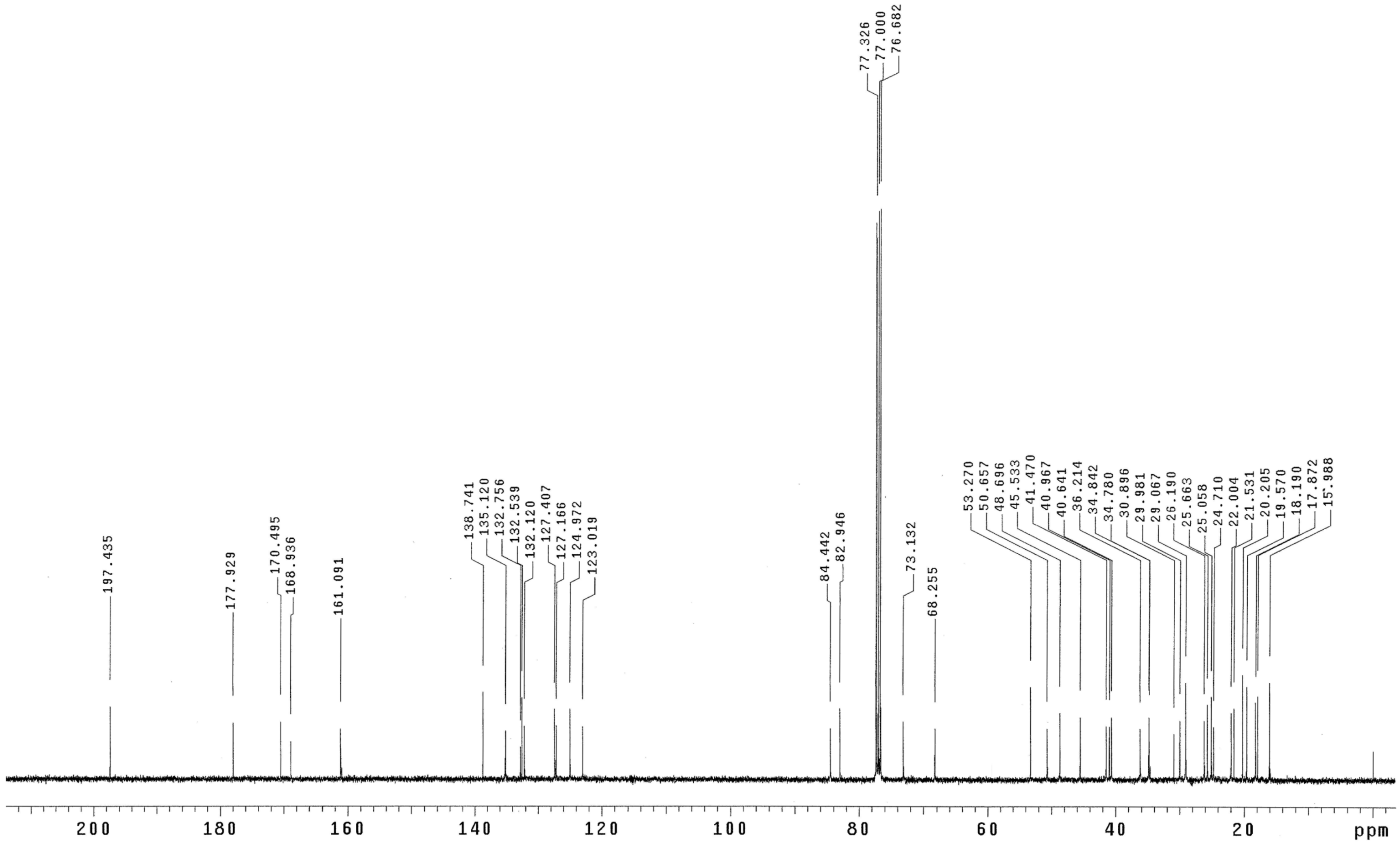

S3. ^{13}C NMR spectrum of **1** in $CDCl_3$

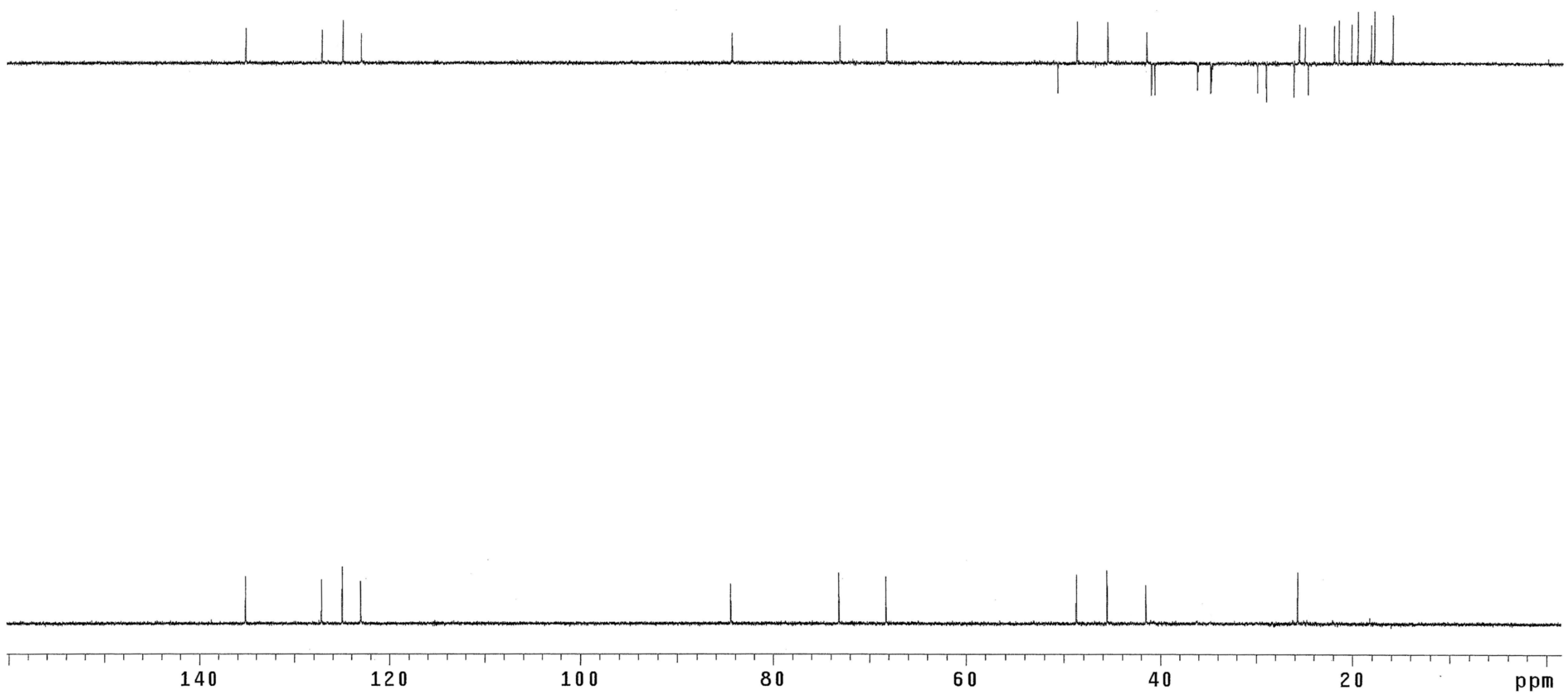

S4. DEPT spectra of **1** in $CDCl_3$

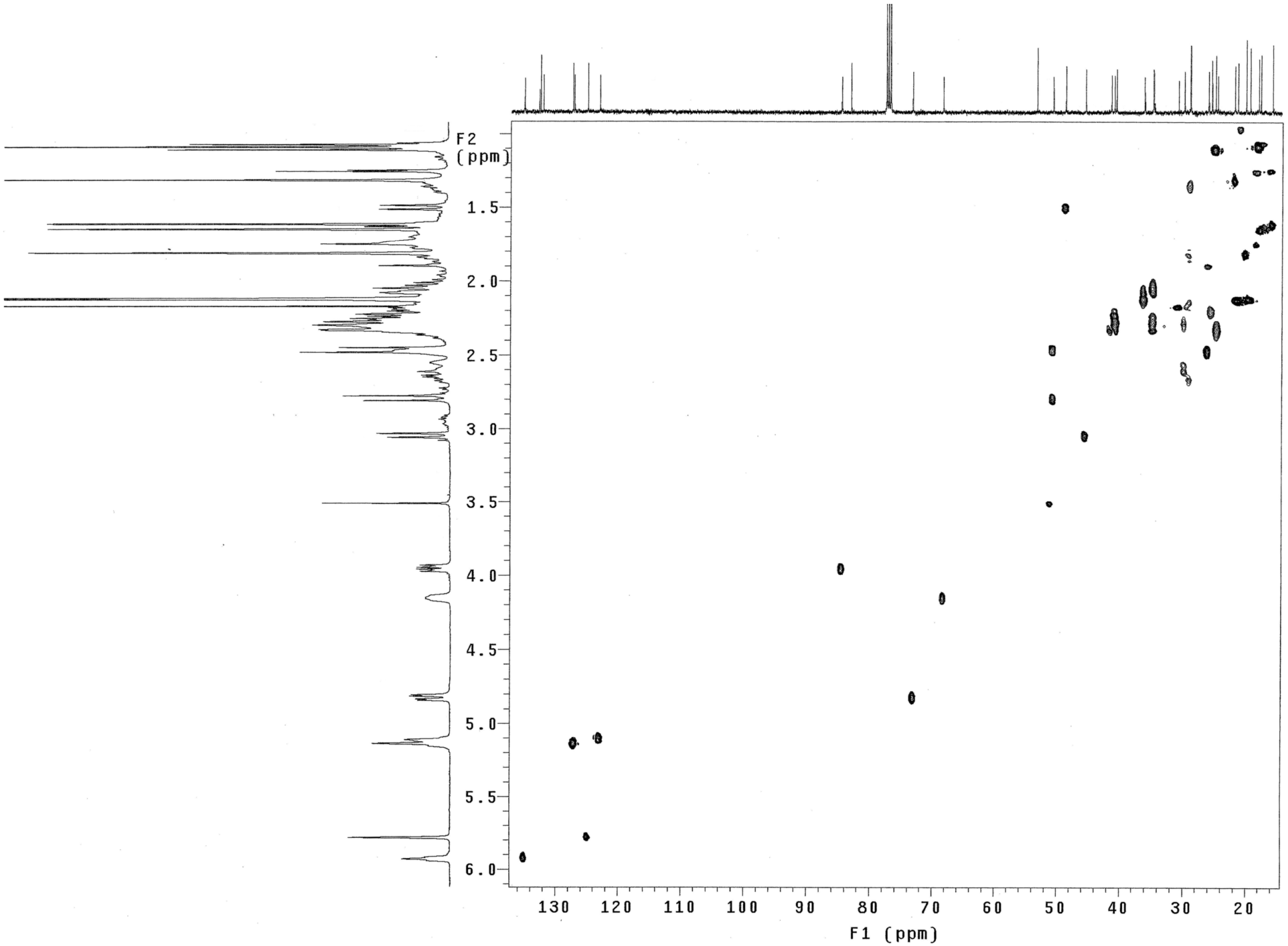

S5. HSQC spectrum of **1** in $CDCl_3$

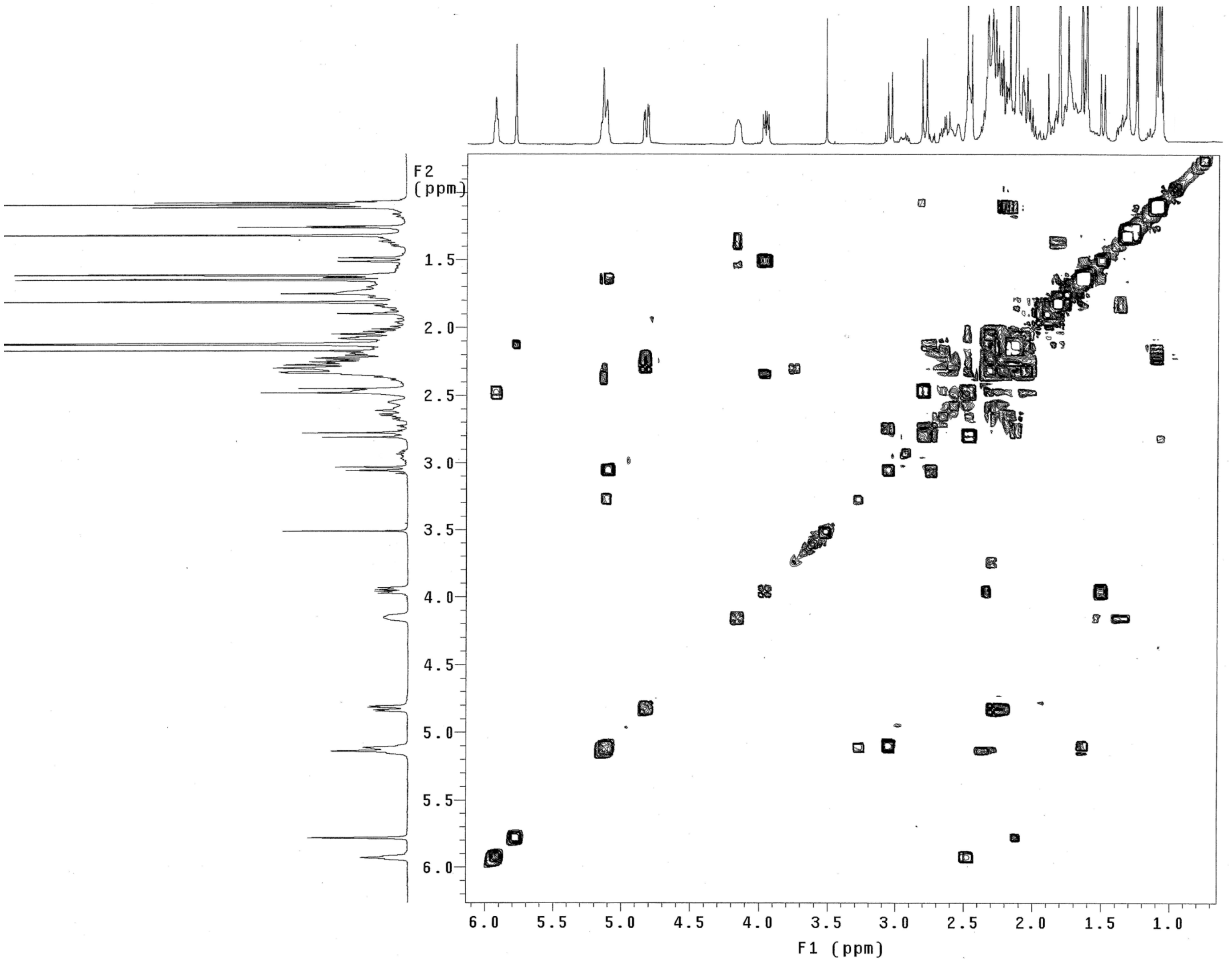

S6. COSY spectrum of **1** in $CDCl_3$

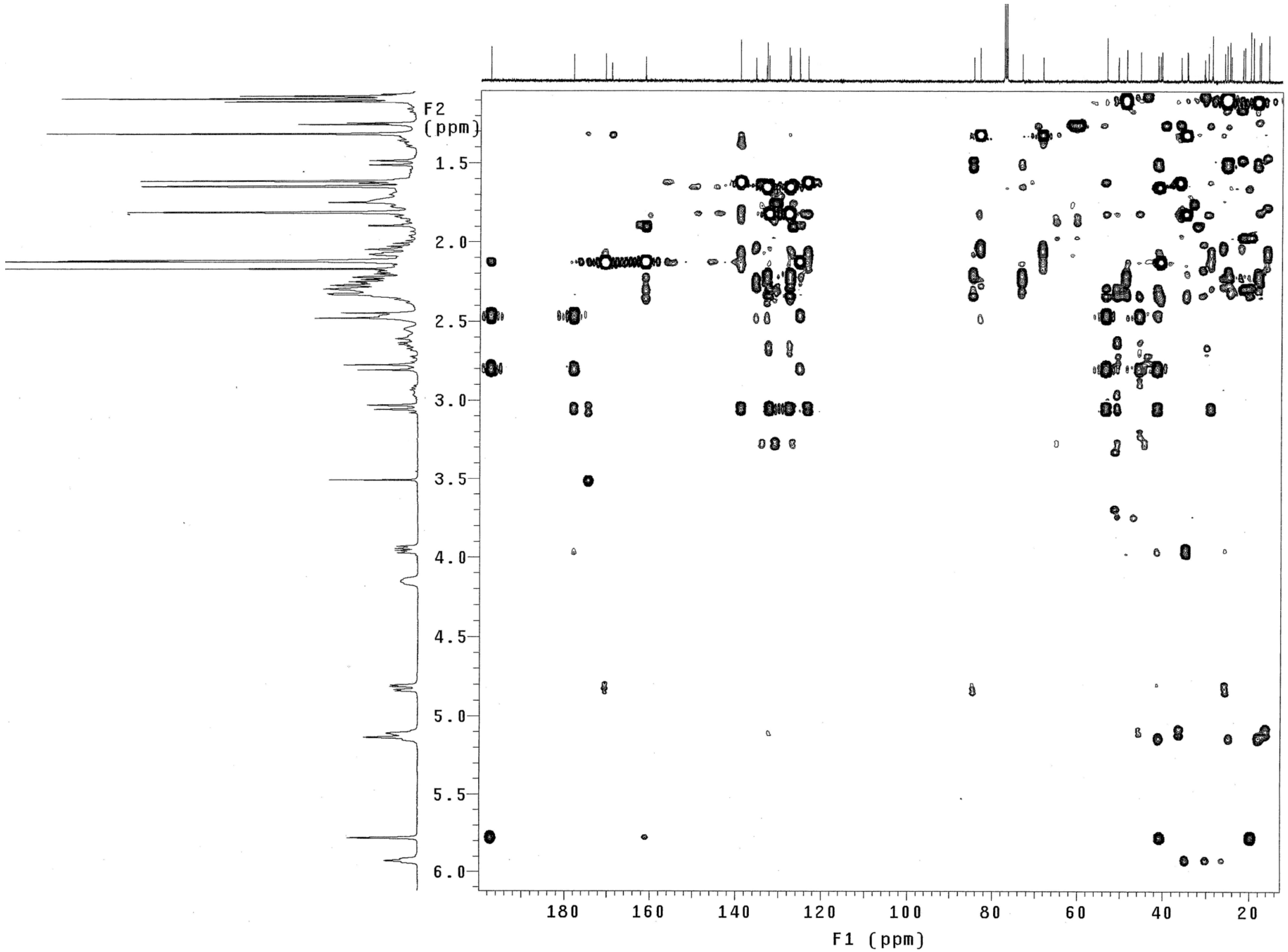

S7. HMBC spectrum of **1** in $CDCl_3$

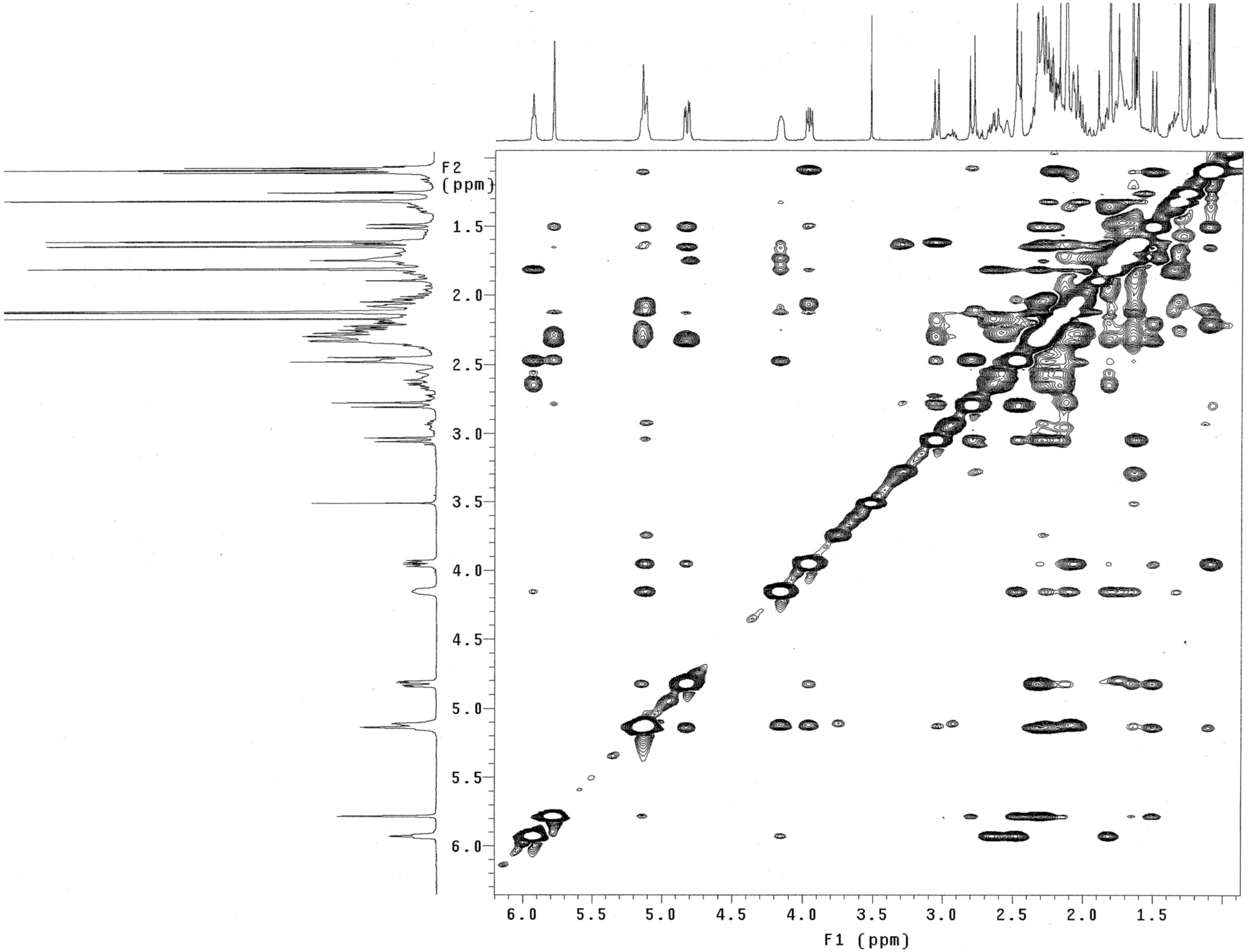

S8. NOESY spectrum of **1** in $CDCl_3$

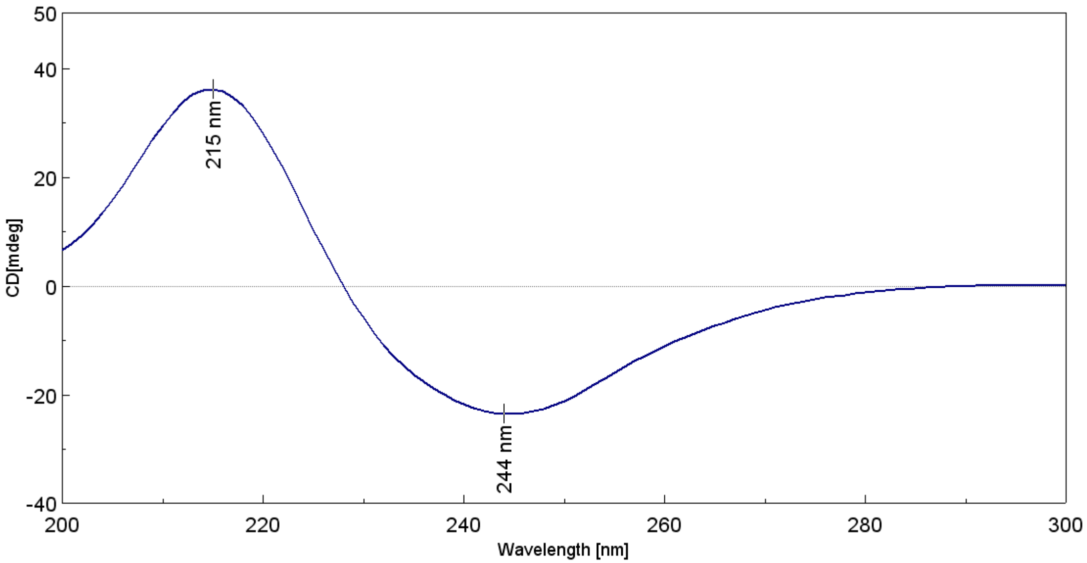

S9. CD spectrum (1.9× 10^{-4}, MeOH) of **1**.

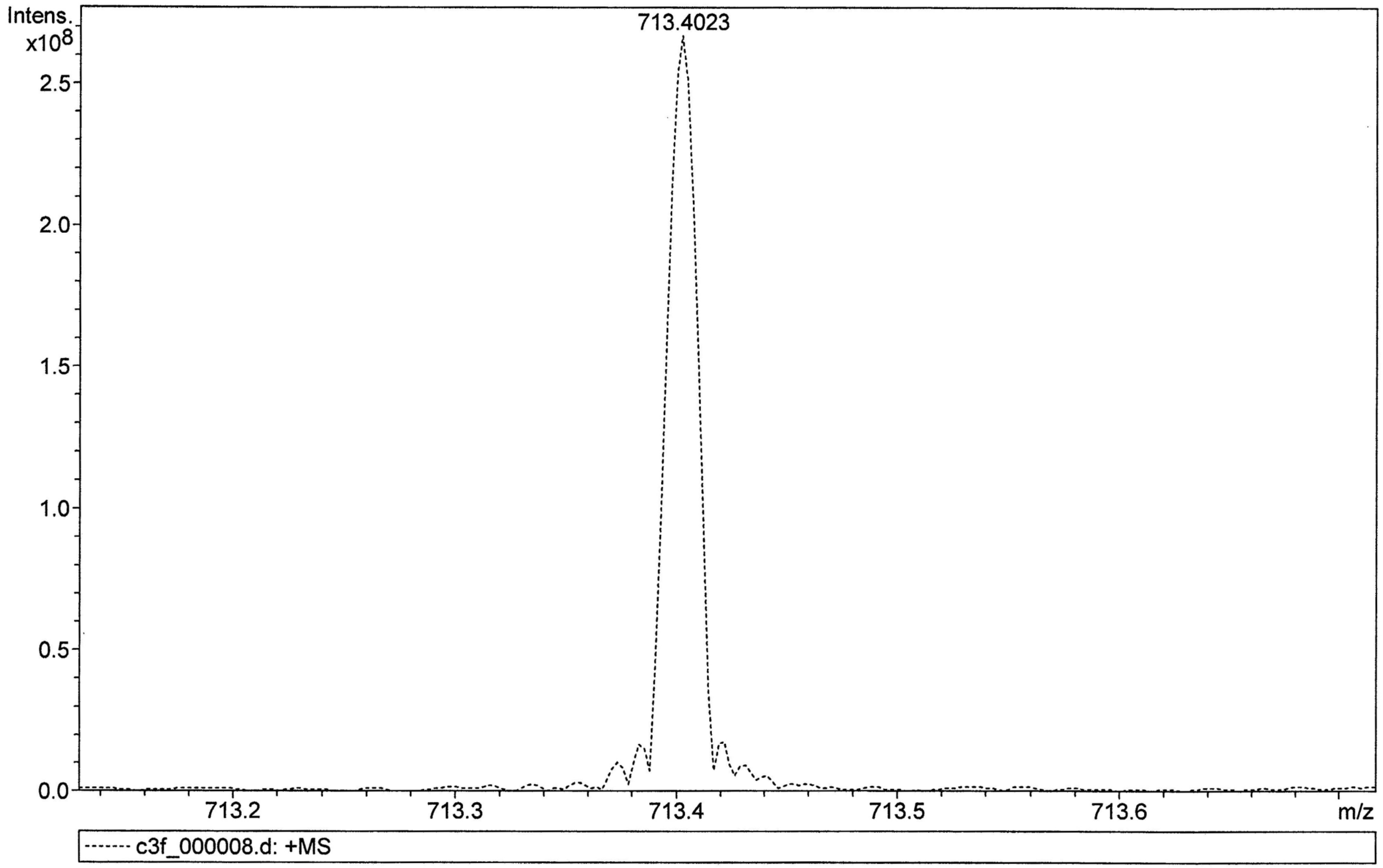

Meas. m/z	#	Formula	Score	m/z	err [mDa]	err [ppm]	mSigma	rdb	e⁻ Conf	N-Rule
713.4023	1	C 42 H 58 Na O 8	100.00	713.4024	0.1	0.1	6.0	13.5	even	ok

S10. HREIMS spectrum of **2**

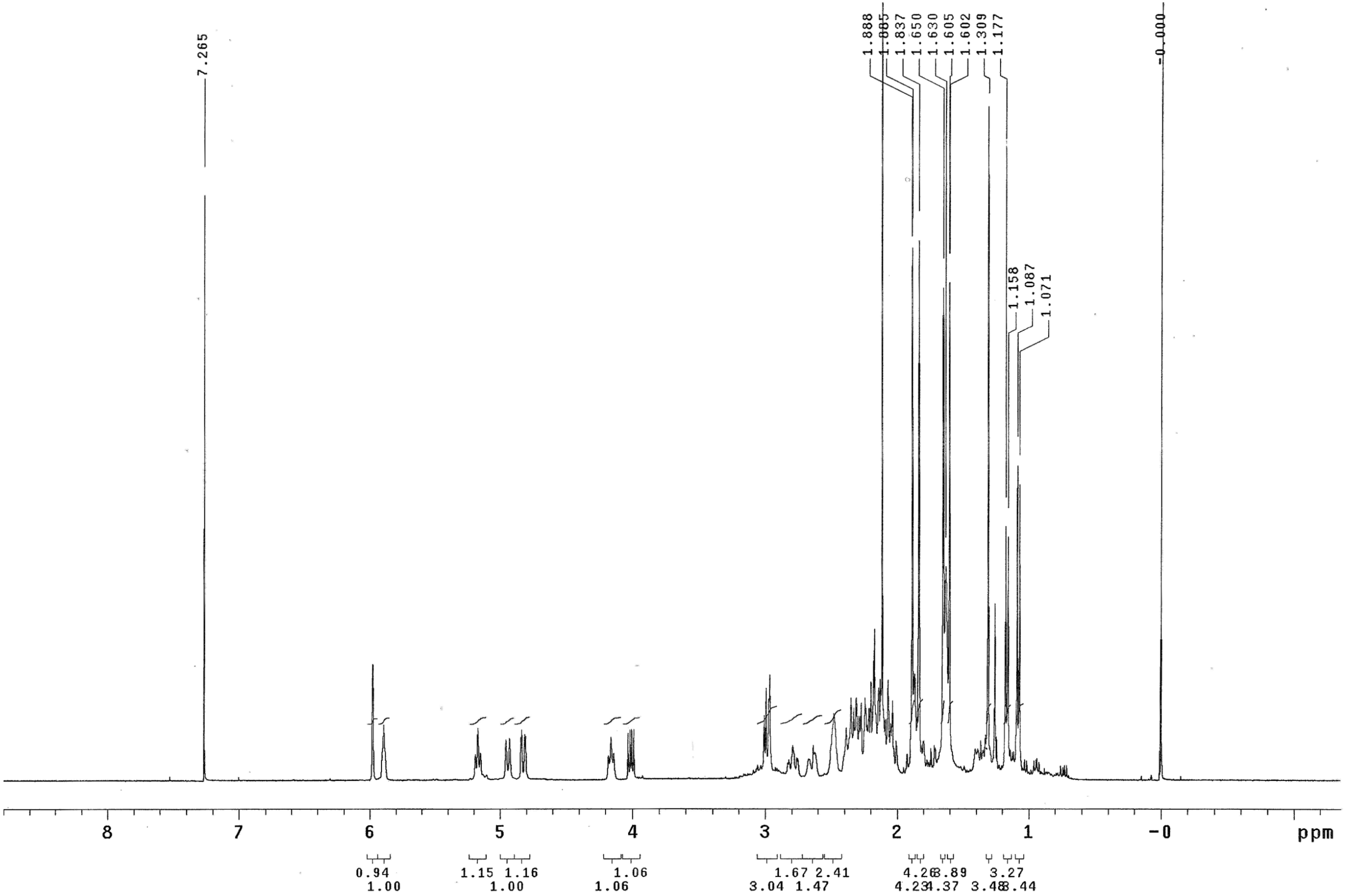

S11. ^{1}H NMR spectrum of **2** in $CDCl_3$

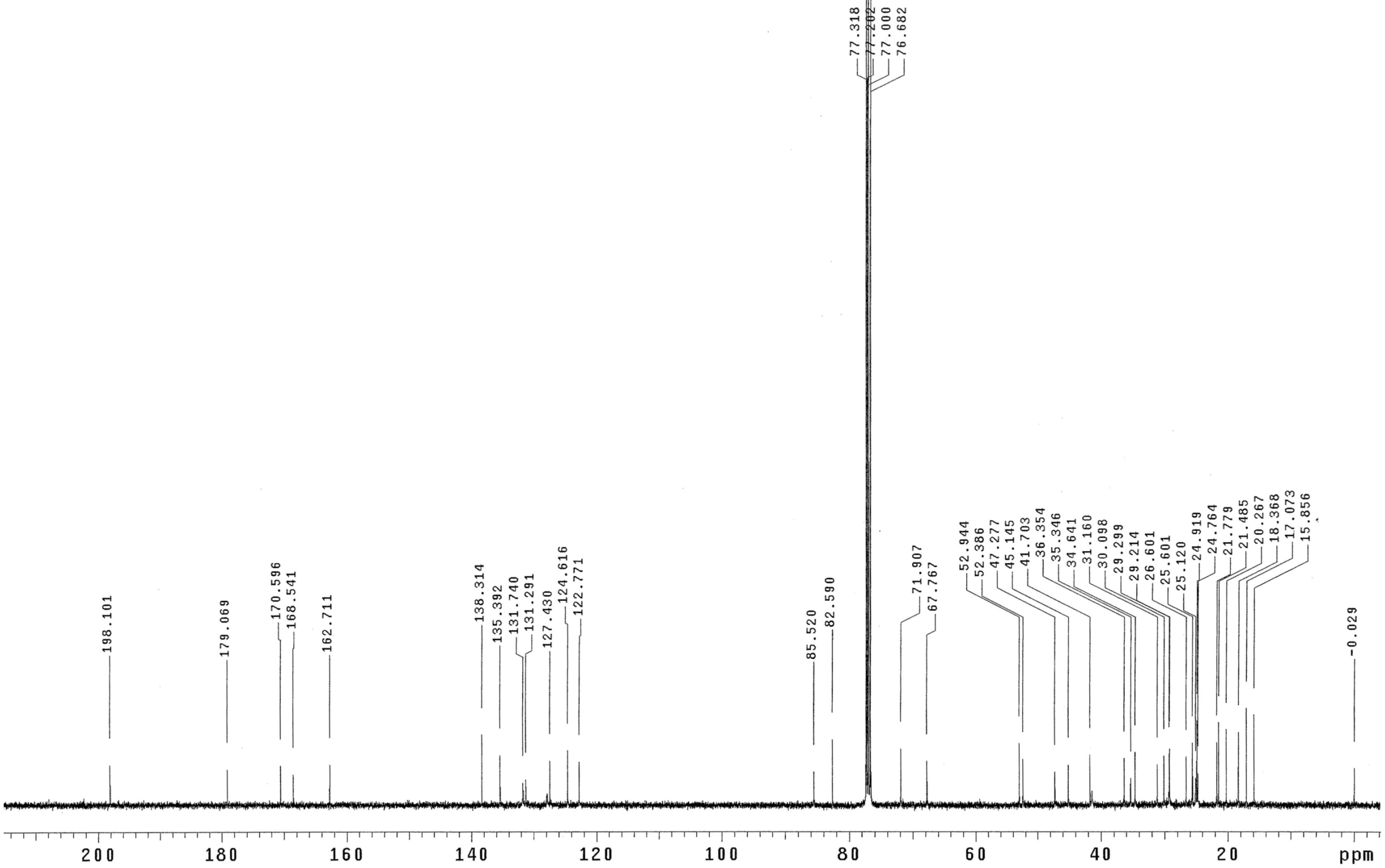

S12. ^{13}C NMR spectrum of **2** in $CDCl_3$

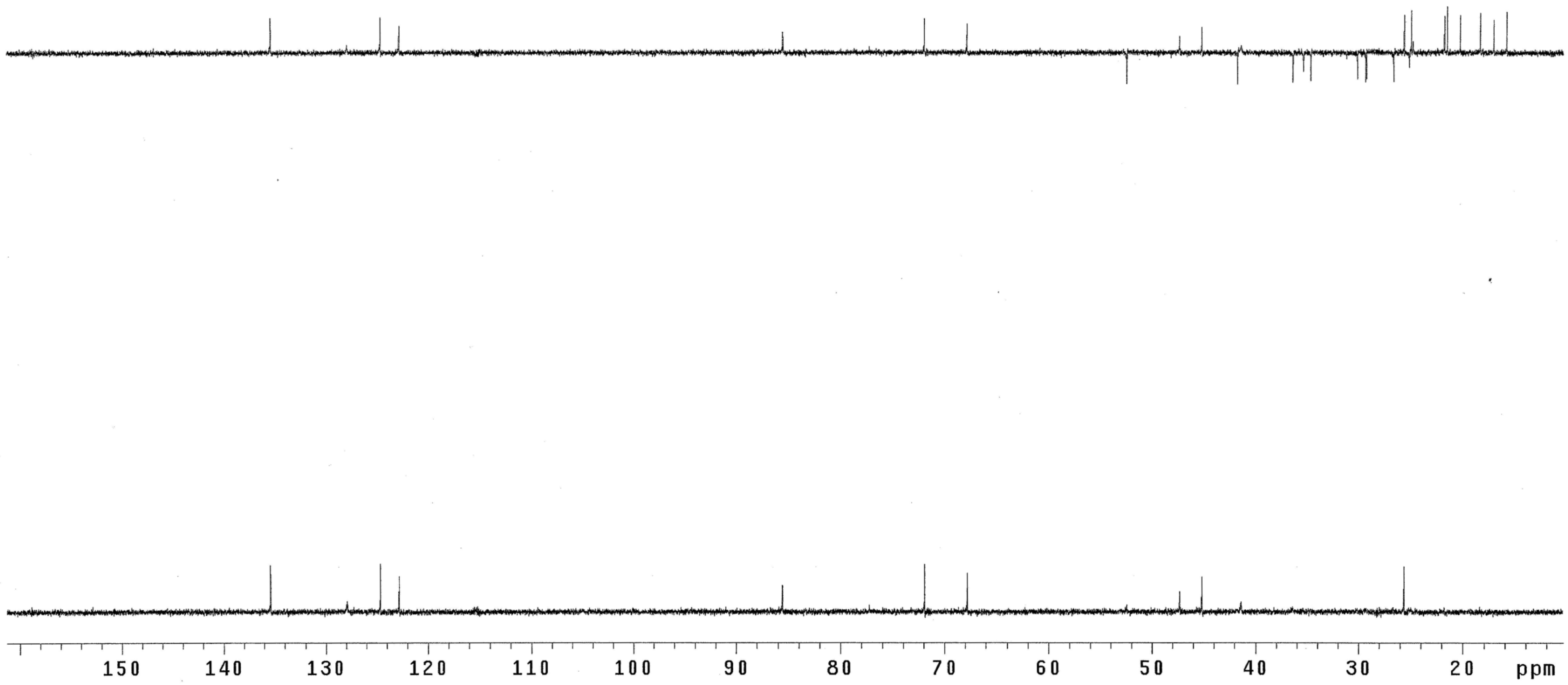

S13. DEPT spectra of **2** in $CDCl_3$

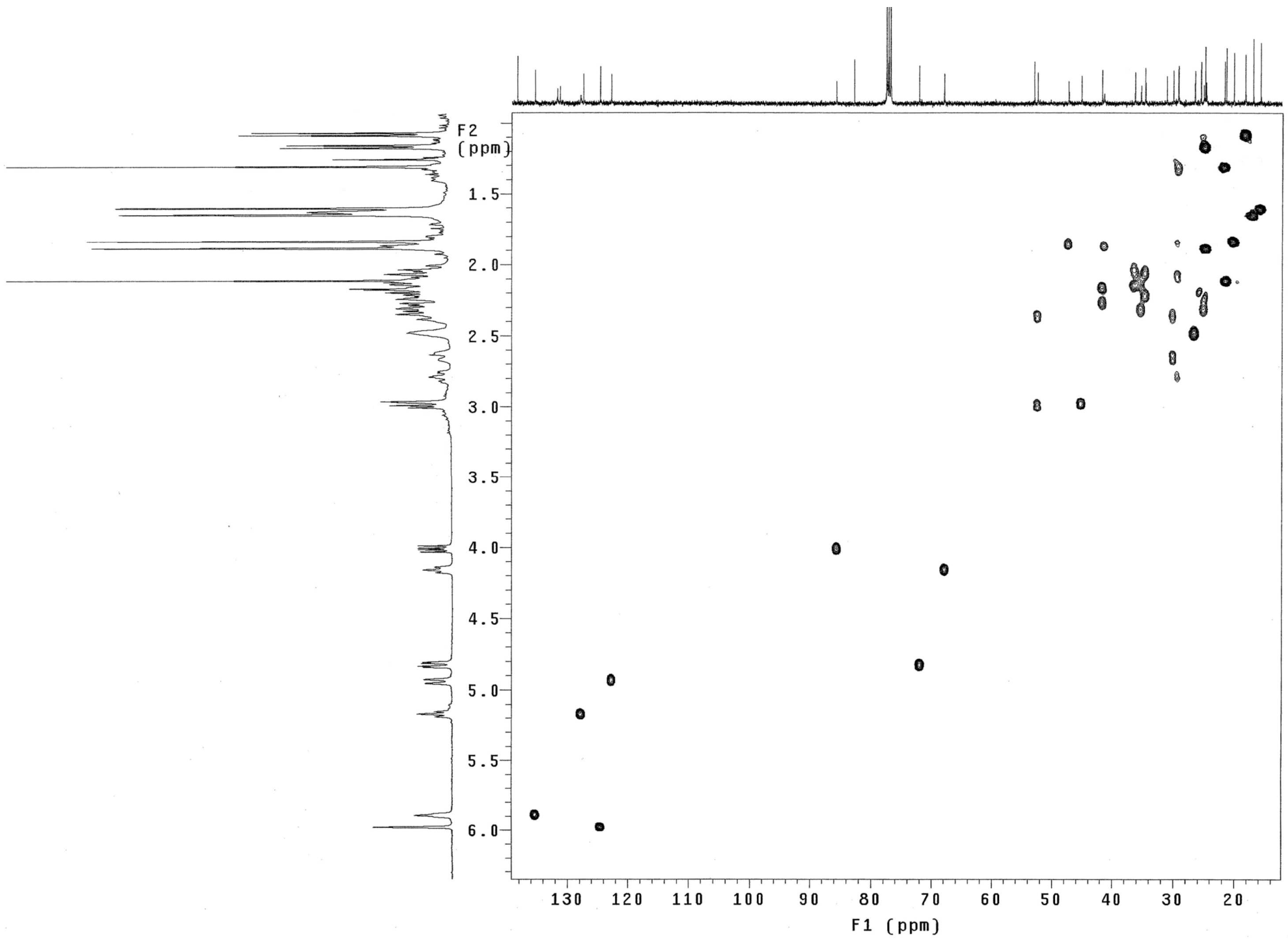

S14. HSQC spectrum of **2** in $CDCl_3$

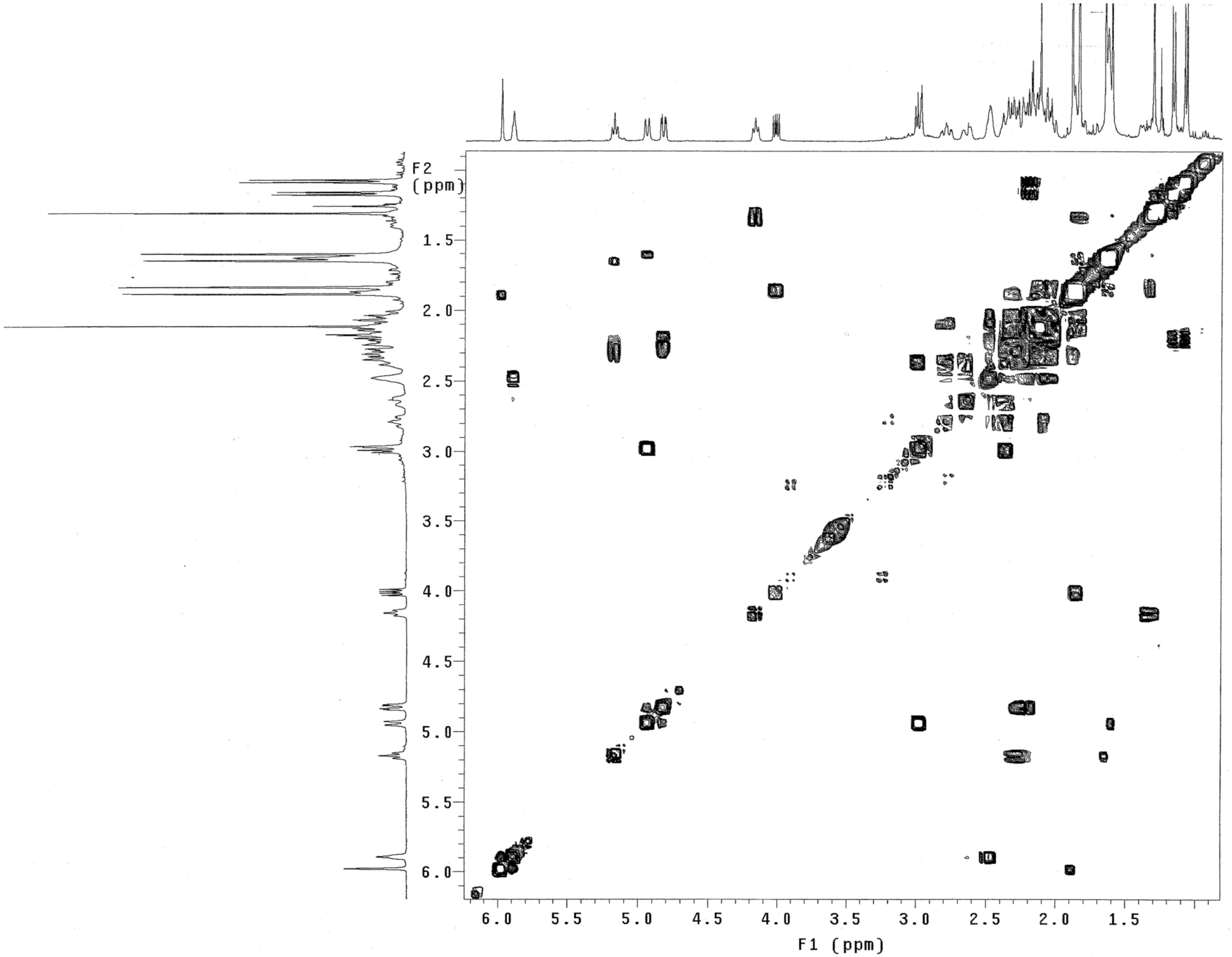

S15. COSY spectrum of **2** in $CDCl_3$

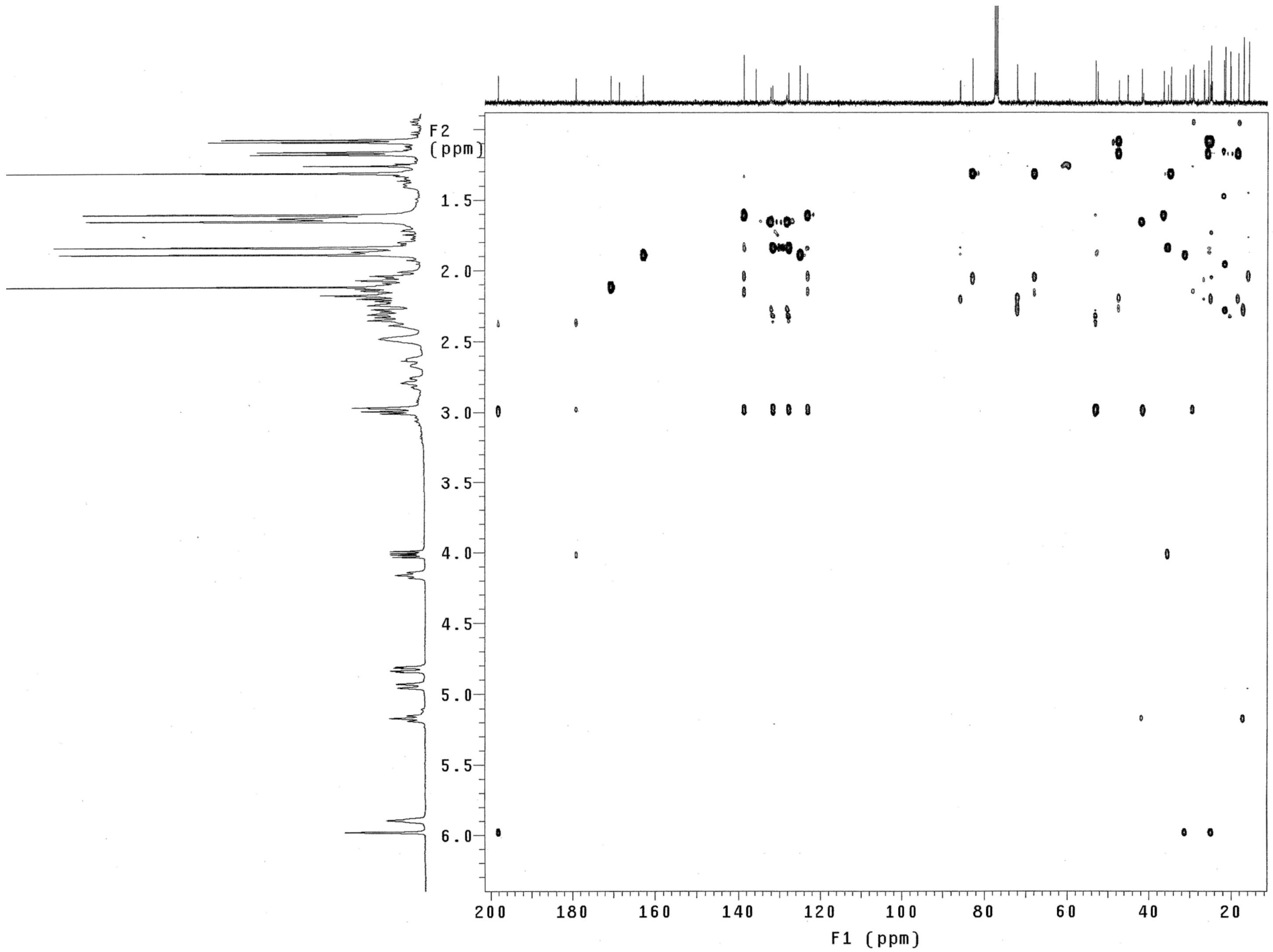

S16. HMBC spectrum of **2** in $CDCl_3$

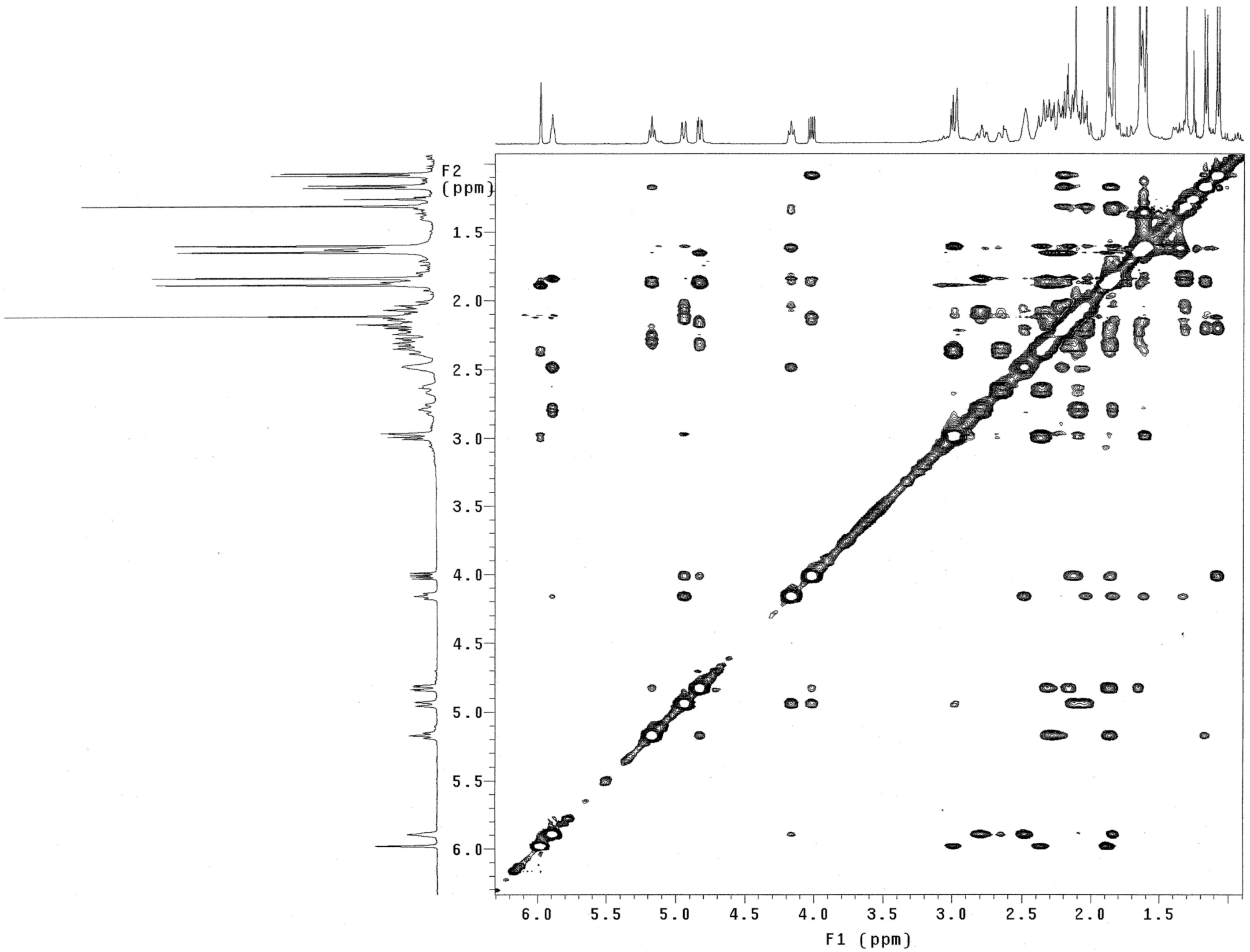

S17. NOESY spectrum of **2** in $CDCl_3$
